# Therapeutic Potential of Ginsenoside Rb1-PLGA Nanoparticles for Heart Failure Treatment via the ROS/PPARα/PGC1α Pathway

**DOI:** 10.3390/molecules28248118

**Published:** 2023-12-15

**Authors:** Lixin Du, Huiling Lu, Ziyan Wang, Chengxin Liu, Yifei Xiao, Zhihua Guo, Ya Li

**Affiliations:** 1 College of Pharmacy, Hunan University of Chinese Medicine, Changsha 410208, China; 20223723@stu.hnucm.edu.cn (L.D.); 20203696@stu.hnucm.edu.cn (H.L.); 20213659@stu.hnucm.edu.cn (Y.X.); 2 College of Chinese Medicine, Hunan University of Chinese Medicine, Changsha 410208, China; 20212011@stu.hnucm.edu.cn (Z.W.); 20212010@stu.hnucm.edu.cn (C.L.); 004294@hnucm.edu.cn (Z.G.)

**Keywords:** ginsenoside Rb1, nanoparticles, heart failure, oxidative stress, ROS/PPARα/PGC1α

## Abstract

(1) Background: Ginsenoside Rb1-PLGA nanoparticles (GRb1@PLGA@NPs) represent a novel nanotherapeutic system, yet their therapeutic efficacy and underlying mechanisms for treating heart failure (HF) remain unexplored. This study aims to investigate the potential mechanisms underlying the therapeutic effects of GRb1@PLGA@NPs in HF treatment; (2) Methods: The left anterior descending coronary artery ligation was employed to establish a HF model in Sprague-Dawley rats, along with an in vitro oxidative stress model using H9c2 myocardial cells. Following treatment with GRb1@PLGA@NPs, cardiac tissue pathological changes and cell proliferation were observed. Additionally, the serum levels of biomarkers such as NT-proBNP, TNF-α, and IL-1β were measured, along with the expression of the ROS/PPARα/PGC1α pathway; (3) Results: GRb1@PLGA@NPs effectively ameliorated the pathological status of cardiac tissues in HF rats, mitigated oxidative stress-induced myocardial cell damage, elevated SOD and MMP levels, and reduced LDH, MDA, ROS, NT-proBNP, TNF-α, and IL-1β levels. Furthermore, the expression of PPARα and PGC1α proteins was upregulated; (4) Conclusions: GRb1@PLGA@NPs may attenuate myocardial cell injury and treat HF through the ROS/PPARα/PGC1α pathway.

## 1. Introduction

Heart failure (HF) refers to the impairment of both the systolic and diastolic functions of the heart, leading to an inadequate ejection of blood and causing insufficient arterial perfusion and circulatory disturbances. HF can be categorized into acute and chronic stages, clinically presenting as dyspnea, arrhythmias, fatigue, and cardiogenic shock [1,2,3]. HF represents the final stage of cardiac diseases, as nearly all cardiovascular conditions eventually culminate in HF occurrence. Factors such as myocardial infarction, excessive hemodynamic load, myocardial hypoxia, and inflammation contribute to cardiac dysfunction [1,4]. With the aggravation of population aging, the prevalence of HF has significantly risen, transforming HF into a pressing public health issue with substantial economic implications [5]. Despite numerous therapeutic approaches for HF, the outcomes remain suboptimal [6]. Therefore, elucidating the underlying mechanisms of HF continues to be a focal point in cardiovascular research.

The pathophysiological underpinnings of HF are characterized by a complex interplay of factors, prominently manifesting as both the structural impairment of the heart and inadequate pumping function [7]. An extensive body of research has underscored that oxidative stress and disrupted energy metabolism are the two primary culprits behind the onset of HF, and intriguingly, these factors are inherently interconnected [8,9,10,11]. Oxidative stress involves an excessive production of reactive oxygen species (ROS) within the biological milieu [12], concomitant with a waning antioxidant defense, thereby instigating a process of oxidative damage to the body’s systems. Through modifications, ROS not only inflict direct harm upon the electrophysiology and the contraction–relaxation dynamics of myocardial cells [13], but also initiate a cascade of hypertrophic signaling kinases and transcription factors, indirectly precipitating myocardial cell hypertrophy [14]. In addition, emerging research suggests that an excess of ROS mediates DNA and mitochondrial damage, and activates pathways that induce myocardial cell apoptosis and engender energy disturbances [15]. Central to these mechanisms, peroxisome proliferator-activated receptor alpha (PPARα), a crucial cell differentiation transcription factor, exhibits robust expression within cardiac tissue and plays a pivotal role in governing myocardial energy metabolism [16]. Concurrently, peroxisome proliferator-activated receptor gamma coactivator 1 alpha (PGC1α) is implicated in the augmentation of mitochondrial biogenesis, the orchestration of mitochondrial equilibrium, and the mitigation of myocardial remodeling [17]. Notably, reports have documented that the activation of PPARα throughout the progression of HF conspicuously augments ATP generation, thereby sustaining normal cardiac function [18,19]. Conversely, mice with PGC1α depletion display symptomatic HF at early stages. The ROS/PPARα/PGC1α pathway emerges as a promising avenue for enhancing myocardial energy metabolism and potentially offering a prospectively promising therapeutic strategy for the treatment of HF.

Ginsenoside Rb1 (GRb1), the most abundant monomeric saponin derived from ginseng, has gained significant attention in recent years for its diverse applications within the cardiovascular domain. Widely investigated in cardiovascular research, GRb1 has been associated with a spectrum of beneficial effects [20,21,22,23], including the enhancement of cardiac function and remodeling, the mitigation of the myocardial damage ensuing from ischemia–reperfusion events, the modulation of the myocardial calcium ion concentration, and the attenuation of myocardial cell apoptosis. Particularly noteworthy is GRb1’s potential to effectively treat HF by orchestrating energy metabolism, rendering it a pivotal ‘hotspot drug’ in the realm of cardiovascular disease research. GRb1@PLGA@NPs, ingeniously engineered as nanoparticles with PLGA as the carrier, harnesses an emulsion solvent evaporation technique to encapsulate GRb1 into diminutive nanoparticles. Our preliminary endeavors [24] have concentrated on the optimization of the fabrication process of GRb1@PLGA@NPs. Through an array of characterization methodologies, we substantiate the successful encapsulation of GRb1 within the PLGA matrix. Moreover, outcomes from in vitro drug release and pharmacokinetic studies underscore the inherent sustained-release capabilities of GRb1@PLGA@NPs, further demonstrating an improved oral bioavailability. These findings collectively underscore the potential of GRb1@PLGA@NPs as a versatile therapeutic entity.

**Figure 1:**
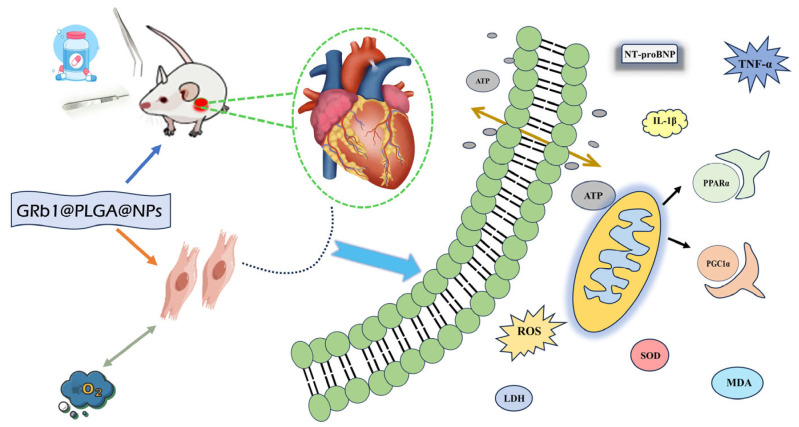
Schematic representation of the study investigating the therapeutic effects of GRb1@PLGA@NPs on HF.

Within the ambit of this investigative study, we embarked on the establishment of an in vitro oxidative stress model employing H9c2 myocardial cells, simulating the repercussions of hypoxia–reoxygenation, in tandem with an in vivo HF model employing Sprague-Dawley (SD) rats. Our primary focus encompassed the evaluation of the protective effects of GRb1@PLGA@NPs against myocardial injury, with a dedicated exploration of the potential mechanisms underpinning GRb1@PLGA@NPs’ therapeutic efficacy through the ROS/PPARα/PGC1α pathway, thereby orchestrating the energy metabolism to mitigate HF. An illustrative depiction of our research framework can be found in Figure 1.

## 2. Results

### 2.1. Preparation of GRb1@PLGA@NPs

**Figure 2:**
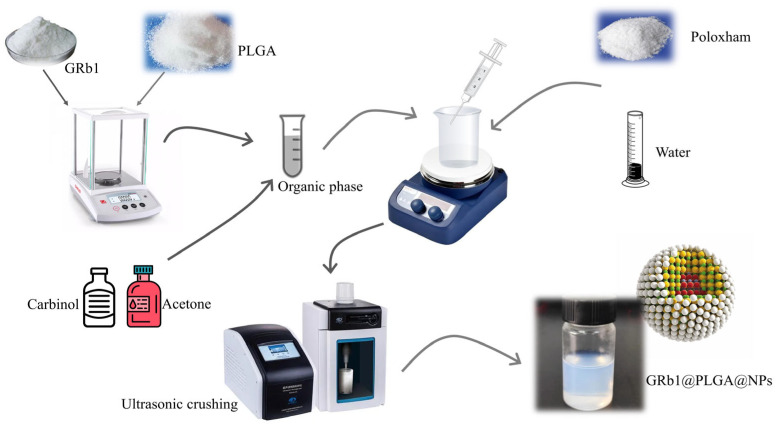
Schematic diagram of the preparation process of GRb1@PLGA@NPs.

The preparation of GRb1@PLGA@NPs was accomplished using an emulsification solvent evaporation method. During this process, when the organic phase containing GRb1 was injected into the aqueous phase, the presence of co-solvent poloxamer facilitated the cross-linking interaction between GRb1 and PLGA as they were stirred. As the methanol and acetone evaporated completely, GRb1 was successfully encapsulated by PLGA. The resulting GRb1@PLGA@NPs exhibit a uniform and stable morphology, characterized by a faint blue opalescence. The nanoparticle size is measured at 120.63 nm, with a low polydispersity index (PDI) of 0.172 and a Zeta potential of −22.67 mV. The preparation process and the final product are depicted in Figure 2.

### 2.2. In Vitro Cytotoxicity

**Figure 3:**
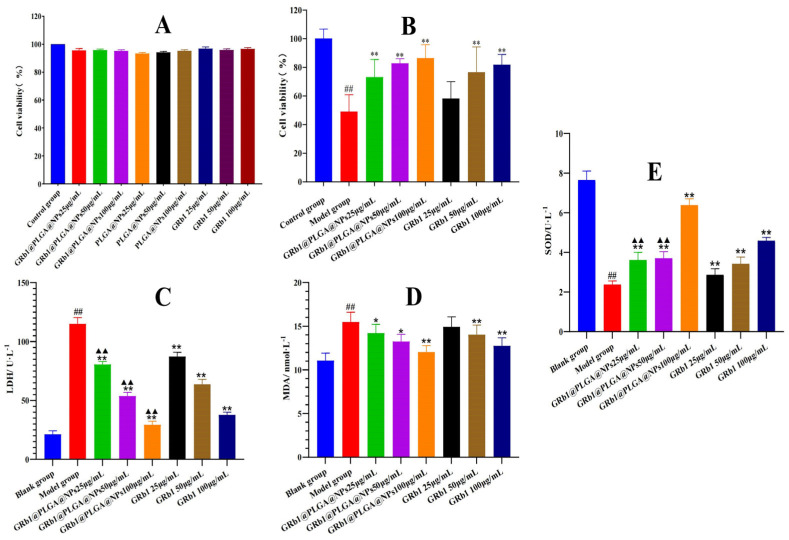
The results of cell viability assessed using the CCK-8 assay (**A**). The impact of GRb1@PLGA@NPs and GRb1 on the survival rate of hypoxia–reoxygenation-treated H9c2 cells (**B**). The effects of GRb1@PLGA@NPs and GRb1 on changes in LDH (**C**), MDA (**D**), and SOD (**E**) levels in hypoxia–reoxygenation-treated H9c2 cells. ^##^ *p* < 0.01 vs. normal group, * *p* < 0.05 and ** *p* < 0.01 vs. model group, ^▲▲^ *p* < 0.01 vs. GRb1.

The in vitro cytotoxicity assay primarily assessed the impact of the substances on cell viability, thereby evaluating their in vivo biocompatibility. As shown in Figure 3A, GRb1@PLGA@NPs, GRb1, and PLGA@NPs exhibited negligible toxicity towards H9c2 cells. The cytotoxicity assay was designed to determine whether the drug affected H9c2 cell survival, so we did not compare GRb1@PLGA@NPs with GRb1. Compared to the control group, the cell viability in different concentration groups remained above 90%. This indicates that the conversion of GRb1 into nanoparticles does not compromise cell vitality. Moreover, the PLGA material displayed no significant interfering effects, indicating favorable biocompatibility.

### 2.3. GRb1@PLGA@NPs Alleviate Hypoxia–Reoxygenation Injury in H9c2 Cells

After the hypoxia–reoxygenation treatment, the survival rate of normally growing H9c2 cells decreased by half. However, when pre-treated with GRb1@PLGA@NPs and GRb1 before undergoing hypoxia–reoxygenation, the cell survival rate remained around 80%, significantly higher than that of the model group. As shown in Figure 3B, with increasing concentration, both GRb1@PLGA@NPs and GRb1 exhibited an enhanced myocardial protective effect. Although there was no significant difference in the high-dose groups of GRb1 and GRb1@PLGA@NPs, the cell survival rate in the GRb1@PLGA@NPs group was 86.39%, while in the GRb1 group, it was 81.73%. The effect on the cell survival rate clearly demonstrates the superiority of GRb1@PLGA@NPs over GRb1. Moreover, the protective effect of different concentrations of GRb1@PLGA@NPs was consistently higher than that of the corresponding concentration of GRb1. This may be attributed to the conversion of GRb1 into nanoparticles, enhancing the absorption of the drug by H9c2 cells.

### 2.4. GRb1@PLGA@NPs Reduced the Intracellular Levels of LDH, MDA and Increased the Content of SOD in Hypoxia–Reoxygenation-Treated H9c2 Cells

In order to further clarify the advantages of GRb1@PLGA@NPs over GRb1, we determined the content changes in the related oxidative stress indexes. Comparatively, hypoxia–reoxygenation-treated H9c2 cells exhibited a significant increase in intracellular LDH, MDA levels and a significant decrease in SOD levels compared to normal cells. However, after treatment with GRb1@PLGA@NPs and GRb1, the intracellular LDH and MDA levels decreased, while the SOD levels increased. Notably, the therapeutic effect of GRb1@PLGA@NPs was superior to that of GRb1 alone. In the high-dose group of the GRb1@PLGA@NPs treatment, the LDH and MDA levels were nearly restored to normal cell levels. The results indicated that the treatment with GRb1@PLGA@NPs was more effective than GRb1, showing significant differences in the content changes in the LDH and SOD indicators, as depicted in Figure 3C–E. Considering the cumulative results of the LDH, MDA, and SOD measurements, we selected the high-dose groups of GRb1@PLGA@NPs and GRb1 for subsequent assessments of ROS and MMP.

### 2.5. GRb1@PLGA@NPs Reduced Intracellular ROS Levels in Hypoxia–Reoxygenation-Treated H9c2 Cells

**Figure 4:**
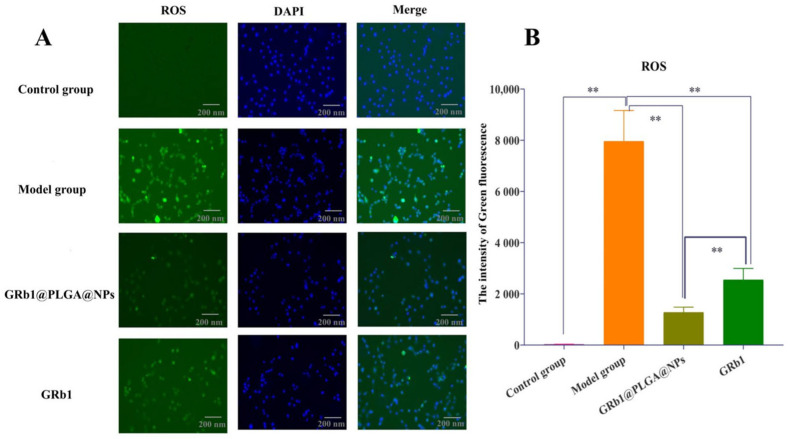
Fluorescence intensity diagram of the effect of GRb1@PLGA@NPs on intracellular ROS in hypoxia–reoxygenated H9c2 cells. Scale bar, 200 nm (**A**). Semi-quantitative Analysis of ROS (**B**). ** *p* < 0.01.

Cellular ROS levels were assessed using the DCFH-DA fluorescence probe. The ROS generated within cells oxidize non-fluorescent DCFH into fluorescent DCF. DAPI was used to stain the cell nuclei, where the ROS fluorescence appears green and DAPI staining appears blue. The results, as shown in Figure 4, reveal that compared to the control group, the model group exhibited significantly enhanced green fluorescence, indicating increased ROS production and oxidative damage following hypoxia–reoxygenation treatment. However, upon treatment with GRb1@PLGA@NPs and GRb1, the intensity of the green fluorescence noticeably decreased, indicating their capacity to inhibit intracellular ROS generation. Moreover, the ROS-reducing effect of GRb1@PLGA@NPs was notably superior to that of GRb1.

### 2.6. GRb1@PLGA@NPs Mitigated the Decrease in MMP and Suppressed Apoptosis in H9c2 Cells

**Figure 5:**
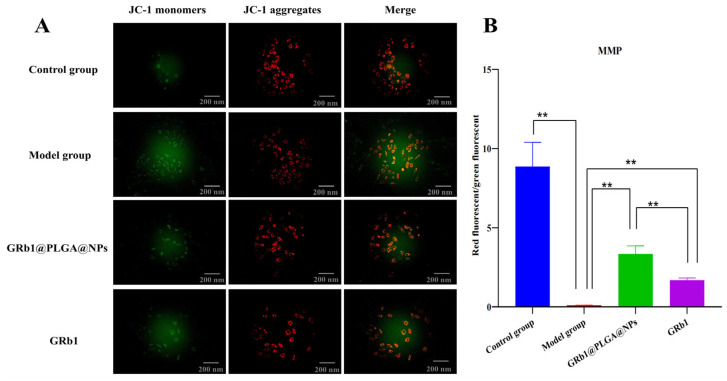
The fluorescence intensity analysis of the effect of GRb1@PLGA@NPs on intracellular MMP in hypoxia–reoxygenation-treated H9c2 cells. Illustrates the fluorescence intensity changes. Scale bar, 200 nm (**A**). Semi-quantitative analysis of MMP (**B**). ** *p* < 0.01.

JC-1 is a fluorescent probe used to detect MMP. Under normal conditions, MMP leads to polymerization and generates red fluorescence, while reduced MMP still generates green fluorescence as monomers. Compared to the blank control group, the model group exhibited significantly increased green fluorescence in intracellular regions, indicating that hypoxia–reoxygenation led to a reduction in MMP in H9c2 cells, resulting in the onset of apoptosis. Compared to the model group, both GRb1@PLGA@NPs and GRb1 interventions led to decreased green fluorescence and increased red fluorescence in intracellular regions, indicating a tendency for MMP elevation and the inhibition of apoptosis through MMP modulation. Moreover, GRb1@PLGA@NPs showed a more pronounced effect in reducing the decline of MMP compared to GRb1. Refer to Figure 5 for details.

### 2.7. Effects of GRb1@PLGA@NPs on the General Condition of HF Rats

**Figure 6:**
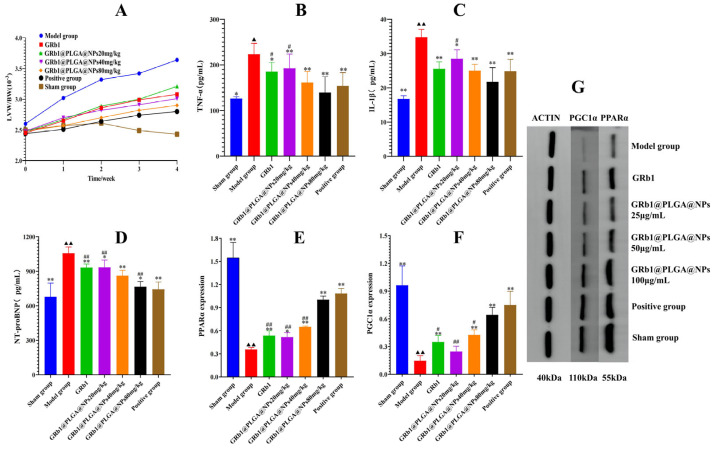
The effects of drug interventions over a period of four weeks on the LVW/BW ratio (**A**). The impact of GRb1@PLGA@NPs on the levels of TNF-α (**B**), IL-1β (**C**), and NT-proBNP (**D**) in the serum of HF rats. The semi-quantitative analysis of PPARα and PGC1α protein expression (**E**,**F**). The influence of GRb1@PLGA@NPs on the protein expression of PPARα and PGC1α in the heart tissue of HF rats (**G**). ^▲^ *p* < 0.05 vs. Sham group, ^▲▲^ *p* < 0.01 vs. Sham group, * *p* < 0.05 vs. Model group, ** *p* < 0.01 vs. Model group, ^#^ *p* < 0.05 vs. Positive group, ^##^ *p* < 0.01 vs. Positive group.

During the administration period, rats in the sham group exhibited a good mental status, reasonable activity, smooth and glossy fur, and minimal aggressive behavior. Rats in the model group displayed dim and disheveled fur, with some fur standing on end. Morning aggressive activities were more frequent and intense, often accompanied by biting. Upon administration of GRb1@PLGA@NPs, the aggressive behavior gradually eased over time, leading to a more docile demeanor and reduced stress. After 4 weeks of treatment, compared to the sham group, rats in the model group showed a significant decrease in body weight. However, all groups of rats receiving treatments exhibited varying degrees of weight gain during the treatment period. The changes in the left ventricular weight index (LVW/BW) of the rats are shown in Figure 6A.

### 2.8. GRb1@PLGA@NPs Improves Histopathological Characteristics in HF Rats

**Figure 7:**
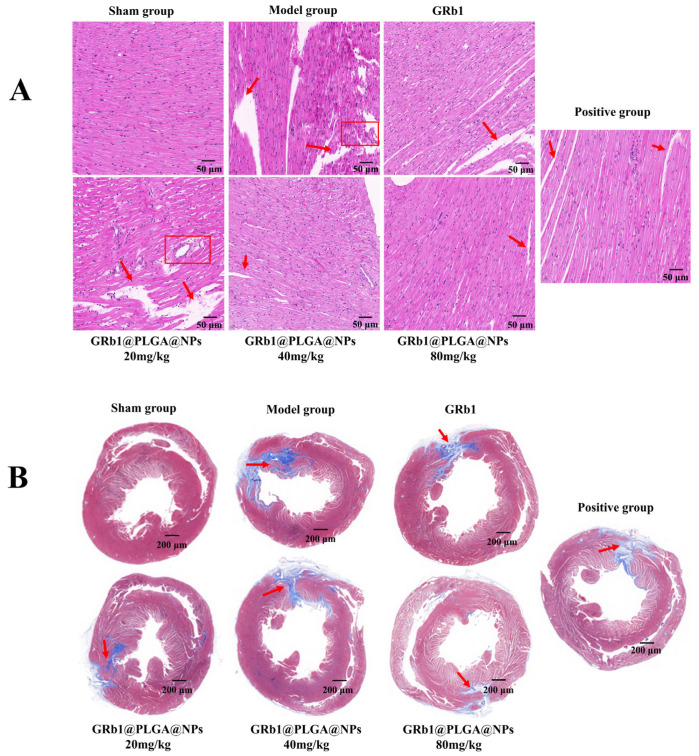
Improvement of pathological histological changes in HF rats by GRb1@PLGA@NPs. HE staining images. Scale bar, 50 μm (**A**). Masson staining images. Scale bar, 200 μm (**B**).

To explore the therapeutic effects of GRb1@PLGA@NPs on HF rats, we prepared histopathological slides of rat myocardial tissues and conducted staining to observe the histopathological changes. The results of the HE staining are presented in Figure 7A. In the sham group, the myocardial tissue of rats displayed a tight and orderly arrangement and an intact cardiomyocyte morphology, and no pathological changes were observed. In the model group, the myocardial tissue exhibited irregular distribution, a disorganized structure, altered cardiomyocyte morphology, increased myocardial gaps, blurred boundaries between various structures, the notable infiltration of inflammatory cells in the myocardial interstitium, and significant fibrous connective tissue proliferation. Compared to the model group, the myocardial tissue damage was somewhat ameliorated in the various treatment groups, as evidenced by the tighter arrangement of myocardial tissue and the restoration of cardiomyocyte morphology.

The Masson’s trichrome staining results (Figure 7B) showed that the myocardial cells in the sham group displayed normal morphology and even distribution, with no significant collagen deposition in the myocardial interstitium, and the compact and regular arrangement of muscle fibers. In the model group, the myocardial gaps were significantly enlarged, muscle fibers were fractured, vacuoles appeared, accompanied by substantial collagen deposition, and myocardial fibrosis tissue increased. After treatment with GRb1@PLGA@NPs and GRb1, the myocardial interstitial gaps were reduced, collagen deposition and muscle fiber fractures were mitigated, and the degree of myocardial fibrosis was alleviated. Notably, the high-dose GRb1@PLGA@NPs group exhibited the best recovery. These results collectively indicate that GRb1@PLGA@NPs effectively ameliorate various histopathological characteristics of myocardial tissue in HF rats.

### 2.9. GRb1@PLGA@NPs Reduce Levels of NT-proBNP, TNF-α, and IL-1β in HF Rats

Compared to the sham group, the model group exhibited a significant increase in the levels of heart failure biomarker NT-proBNP, as well as the pro-inflammatory cytokines TNF-α and IL-1β. In comparison to the model group, treatment with GRb1@PLGA@NPs, GRb1, and carvedilol led to a significant reduction in the levels of NT-proBNP, TNF-α, and IL-1β. Among these treatments, the TNF-α levels approached those of the normal rats, and the effects of GRb1@PLGA@NPs were superior to those of GRb1 and carvedilol. The results are presented in Figure 6B–D.

### 2.10. GRb1@PLGA@NPs Upregulate the Protein Expression of PPARα and PGC1α, Promoting Energy Metabolism in HF Rats

**Figure 8:**
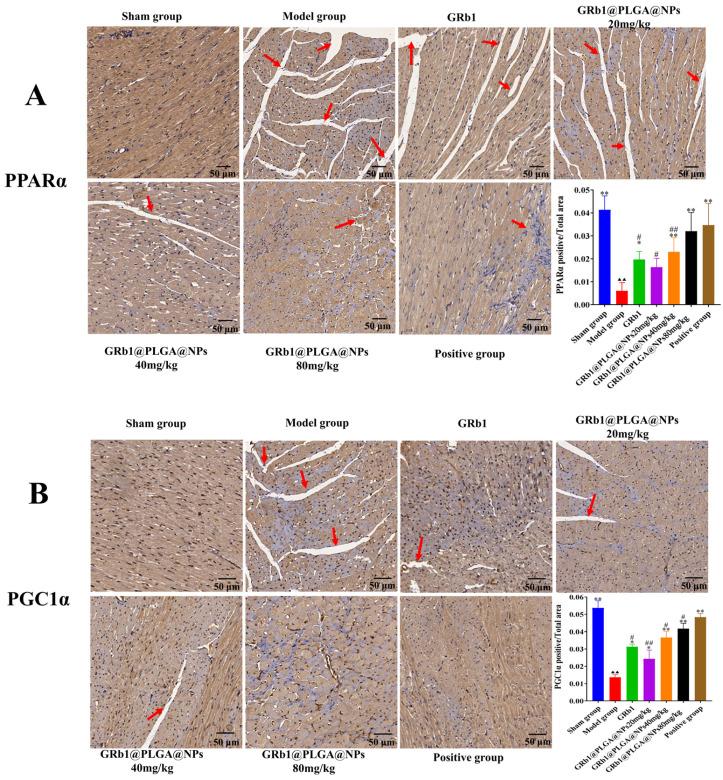
Upregulation of PPARα and PGC1α protein expression in myocardial tissues of HF rats by GRb1@PLGA@NPs. Immunohistochemical staining of PPARα protein. Scale bar, 50 μm (**A**) and PGC1α protein. Scale bar, 50 μm (**B**). ^▲▲^ *p* < 0.01 vs. Sham group, * *p* < 0.05 vs. Model group, ** *p* < 0.01 vs. Model group, ^#^ *p* < 0.05 vs. Positive group, ^##^ *p* < 0.01 vs. Positive group.

The protein expression levels of PPARα and PGC1α in the myocardial tissues are shown in Figure 6E–G. Compared to the sham group, the protein content of PPARα and PGC1α was significantly decreased in the HF model group. However, after treatment with GRb1@PLGA@NPs, GRb1, and trimetazidine, the protein expression of PPARα and PGC1α was upregulated. In comparison to trimetazidine, the therapeutic effect of GRb1@PLGA@NPs showed no significant difference. Immunohistochemical analysis also revealed that compared to the sham group, the protein content of PPARα and PGC1α was decreased in the model group. After treatment, the protein expression of PPARα and PGC1α was significantly increased in all treated groups, as shown in Figure 8.

## 3. Discussion

HF is a prevalent clinical syndrome with high morbidity and mortality rates. During HF, continuous cardiac overload leads to an imbalance between mitochondrial energy supply and demand in cardiomyocytes, resulting in cellular injury and apoptosis [25]. Drug therapy is a primary and widely used approach for HF treatment. Identifying drugs with significant efficacy and minimal side effects is crucial. GRb1 emerges as a promising candidate for HF treatment. Studies have shown that GRb1 improves structural and metabolic disturbances in HF rats, inhibits myocardial fibrosis, and enhances cardiac energy metabolism by suppressing the abnormal upregulation of FADD and increasing ATP generation [26]. Additionally, another study reported that GRb1 mitigates cardiac hypertrophy induced by angiotensin II and directly inhibits the expression of pro-hypertrophic mRNA in response to lipopolysaccharide (LPS) stimulation, thereby reducing inflammation and alleviating HF caused by myocardial hypertrophy [27]. Furthermore, HF rats treated with GRb1 exhibit improved cardiac functions, likely attributed to the activation of the PI3K/mTOR pathway [28].

In light of the significant therapeutic potential of GRb1 for HF, we aimed to enhance its utility by formulating it into nanoparticles. Nanoparticles are useful in nanodelivery system [29], which have gained widespread use in pharmaceutical research in recent years. The reduction in particle size and improved solubility achieved by nanoparticle formation can partially counteract drug elimination, thereby extending the half-life and circulation time in vivo. This enhancement in drug absorption significantly improves bioavailability [30,31,32]. Consequently, we prepared GRb1@PLGA@NPs and established an in vitro oxidative stress model using H9c2 cardiomyocytes, as well as an in vivo HF model in SD rats. By assessing changes in various indices and protein expression, we investigated the mechanism through which GRb1@PLGA@NPs modulate myocardial energy metabolism and treat HF.

H9c2 cardiomyocytes, derived from rat myocardial tissue, offer high physiological relevance, resembling the in vivo environment. They exhibit various characteristics of cardiac muscle cells, including myofibril formation, the expression of cardiac troponins, and contraction properties, making them widely used in cardiovascular disease research. Oxidative stress, a major factor in HF progression, is known to impact apoptosis and physiological changes in H9c2 cells [33]. Intracellular oxidative stress occurs under redox imbalance, triggered by hypoxia or hyperoxia, leading to cell apoptosis. Oxidative stress promotes excessive ROS generation, disrupting cell signaling pathways through the deactivation of phosphatases (PTPIB, PTEN), the hyperphosphorylation of protein tyrosine kinases (PTK), the upregulation of growth-inhibitory proteins, and impaired transcription factor-mediated cell signaling. These events ultimately cause mitochondrial dysfunction, cardiomyocyte apoptosis, and contribute to HF [34,35]. In our study, we induced oxidative stress in H9c2 cells using hypoxia–reoxygenation, and following intervention with GRb1@PLGA@NPs, we observed reduced ROS production, increased SOD levels, decreased LDH and MDA levels, and suppressed MMP decline. SOD, MDA, and LDH are pivotal markers of oxidative stress and antioxidant capacity, while MMP reduction signifies early apoptosis [36]. This indicates that GRb1@PLGA@NPs mitigate hypoxia–reoxygenation-induced oxidative stress by diminishing ROS production, thereby safeguarding cardiomyocytes and inhibiting cell apoptosis.

In vivo animal experiments demonstrated that HF rats treated with GRb1@PLGA@NPs exhibited recovery in pathological features. HE and Masson staining revealed reduced collagen deposition and myofibril disruption in cardiac cells, resulting in diminished myocardial fibrosis. The measurement results indicated a significant decrease in serum NT-proBNP, TNF-α, and IL-1β levels, normalizing their concentrations. Western blotting and immunohistochemistry revealed activated PPARα and PGC1α pathways with upregulated protein expression, suggesting that GRb1@PLGA@NPs alleviate myocardial injury through PPARα and PGC1α mediation. NT-proBNP is a valuable biomarker for HF diagnosis and monitoring, playing a critical role. It contains multiple biologically active peptides, minimally influenced by agents like brain natriuretic peptide inhibitors or recombinant nesiritide. Elevated NT-proBNP levels indicate potential HF occurrence [37,38]. TNF-α and IL-1β are typical inflammatory factors intricately linked to various aspects such as immunity, inflammation, and metabolism, closely associated with HF onset [39]. Relevant analyses have shown a positive correlation between TNF-α, IL-1β, and NT-proBNP expression [40], further affirming the significance of TNF-α and IL-1β in HF detection.

Notably, PPARα and PGC1α appear to be crucial transcription factors regulating cardiac energy metabolism and cell apoptosis. PPARα induces the synthesis of fatty acid transport proteins, fostering oxidative metabolism and serving as a pivotal signaling molecule controlling myocardial mitochondrial capacity. Research indicates that under heavy cardiac load, PPARα expression decreases [41,42], yet its activation aids in maintaining myocardial high-energy phosphate metabolism, reducing glucose oxidation, and exerting protective effects on the myocardium by binding to transcription factors like NF-κB and AP-1 even after ligand binding. PGC1α, a coactivator of PPARα, enhances the transcription of P300 and downstream effector factor MCAD upon binding to PPARα and promotes mitochondrial autogenesis and energy metabolism, thus preserving mitochondrial homeostasis in cardiac cells [43]. Therefore, the activation of the PPARα/PGC1α pathway not only fosters ATP generation, but also regulates cardiac cell metabolism disruption, contributing to the maintenance of normal cardiac function. Our study effectively confirms this mechanism.

## 4. Materials and Methods

### 4.1. Materials

Ginsenoside Rb1 was procured from Nanjing Dilger Medical Technology Co., Ltd. (Nanjing, China). Trypsin, DMEM, FBS, PBS, and RIPA lysis buffer were acquired from Wuhan Promega Biotech Co., Ltd. (Wuhan, China). The CCK-8 assay kit was obtained from Beijing Boao Sen Biotechnology Co., Ltd. (Beijing, China). The LDH, SOD, and MDA assay kits were purchased from Nanjing Jiancheng Bioengineering Institute. (Nanjing, China). The JC-1, ROS, and DAPI staining kits were sourced from Shanghai Beyotime Biotechnology Co., Ltd. (Shanghai, China). PPARα and PGC1α proteins, protease inhibitors, and loading buffer were obtained from Wuhan Sanying Biotechnology Co., Ltd. (Wuhan, China). HRP-conjugated goat anti-mouse secondary antibody, the ECL chemiluminescence kit, TNFα, NT-proBNP, IL-1β, and the BCA protein assay kit were purchased from Wuhan Sibbiotech Inc. (Wuhan, China). Trimetazidine was acquired from Hankang Pharmaceutical Biotechnology Co., Ltd. (Changsha, China). GRb1@PLGA@NPs were synthesized in-house at the laboratory.

### 4.2. Preparation of GRb1@PLGA@NPs

A total of 3.6 mg of GRb1 and 18 mg of PLGA were weighed and dissolved in 1 mL of acetone and 1 mL of methanol, forming the organic phase. Additionally, 10 mL of aqueous phase containing 0.14% pluronic was prepared. The organic phase was gradually introduced into the aqueous phase while subjected to continuous magnetic stirring at 600 rpm and maintained at 30 °C. After 2 h of mixing, the resultant mixture was subsequently sonicated for 10 min using an ultrasonic cell disruptor. The prepared GRb1@PLGA@NPs were characterized by measuring their particle size, polydispersity index (PDI), and zeta potential.

### 4.3. Animal Experiment

A total of 42 male SPF-grade SD rats with an initial weight of (200 ± 20) g were obtained from Hunan Slake Jingda Experimental Animal Co., Ltd., with certificate number ZS-202211150001. These rats were housed in the Animal Experimental Center of Hunan University of Chinese Medicine. The animal housing conditions maintained a temperature of (25 ± 2) °C, relative humidity of (50 ± 4)%, and a 12 h light–dark cycle. They had ad libitum access to food and water. All animal procedures were conducted following the approval of the Animal Ethics Committee of Hunan University of Chinese Medicine, with ethics number LLBH-202212060002. After a 7-day acclimatization period, 36 rats were selected to establish a HF model using the left anterior descending coronary artery ligation, and this was sustained for 6 weeks. Preoperative and postoperative electrocardiograms were observed to confirm the successful establishment of the HF model. The remaining 6 rats served as the sham-operated group (abbreviated as ‘sham group’). The rats in the successfully modeled group were randomly divided into various groups: model group, GRb1@PLGA@NPs groups (20, 40, 80 mg·kg^−1^), GRb1 group (40 mg·kg^−1^), and trimetazidine group (2.03 mg·kg^−1^, abbreviated as ‘positive group’). Rats in the sham and model groups were orally administered an equivalent volume of normal saline daily for 4 weeks.

### 4.4. Cell Culture and Processing

The H9c2 cell line was procured from the Shanghai Cell Bank of the Chinese Academy of Sciences. Cells were cultured in high-glucose DMEM supplemented with 10% FBS and 1% (*v*/*v*) penicillin/streptomycin. The cell cultures were maintained in a CO_2_ incubator at 37 °C with a humidified atmosphere containing 5% CO_2_. Upon reaching 80–90% confluence, cells were passaged. Well-growing cells were divided into various experimental groups: blank control group, model group (subjected to 6 h of hypoxia at 94% N_2_, 5% CO_2_, and 1% O_2_ in a tri-gas incubator, followed by 2 h of reoxygenation in a CO_2_ incubator), GRb1@PLGA@NPs groups (25, 50, 100 μg·mL^−1^, treated with the nanoparticles for 1 h prior to hypoxia–reoxygenation), and GRb1 groups (25, 50, 100 μg·mL^−1^, also treated with GRb1 for 1 h prior to hypoxia–reoxygenation).

### 4.5. Cytotoxicity Assay

Logarithmically growing cells were seeded at a density of 1 × 10^5^/mL into a 96-well plate, with 100 μL per well, and allowed to adhere by growing along the walls in a cell culture incubator. After 24 h, cells were incubated with various concentrations of GRb1@PLGA@NPs, GRb1, and PLGA@NPs (without GRb1) for 24 h. Subsequently, 10 μL of CCK-8 solution was added to each well, and the plates were incubated in the cell culture incubator for 1 h. After incubation, the absorbance values of each group were measured at 450 nm using a microplate reader, and the cell viability was calculated.

### 4.6. The Protective Effects of GRb1@PLGA@NPs on Hypoxia–Reoxygenation Injured Cardiomyocytes

Different concentrations of GRb1@PLGA@NPs and GRb1 were pre-added to a 96-well plate and co-incubated with cells for 1 h. Subsequently, the cells were subjected to a 6 h anaerobic incubation within an anaerobic culture chamber, followed by a 2 h reoxygenation period. Afterward, 10 μL of CCK-8 solution was added to each well, and the plates were incubated in the cell culture incubator for 1 h. The absorbance values of each group were measured at 450 nm using a microplate reader. This investigation aimed to elucidate the protective effects of GRb1@PLGA@NPs on hypoxia–reoxygenation injured cardiomyocytes.

### 4.7. Determination of LDH, SOD and MDA Content

The cells were seeded into 6-well plates with a cultivation volume of 2 mL per well. Subsequently, the cells from each group were collected via centrifugation at 1000 rpm for 4 min. The supernatant from the collected cells was then processed according to the instructions provided with the respective assay kits. Notably, the levels of LDH and MDA were determined using colorimetric assays, while the SOD levels were evaluated using the WST-1 method.

### 4.8. Determination of ROS Content

When the cell growth density reached 80%, grouping was initiated. Following the designated treatments, cells were washed twice with PBS. Subsequently, each well was supplemented with 800 μL of a 10 μM DCFH-DA solution. The plate was then incubated at 37 °C in a light-protected environment for 30 min. The supernatant was discarded, and cells were rinsed twice with pre-cooled PBS. Next, 1 mL of serum-free DMEM was added to each well. The generation of ROS was immediately assessed using fluorescence microscopy.

### 4.9. Changes in Mitochondrial Membrane Potential (MMP)

Similarly, when the cell growth density reached 80%, grouping was initiated. Following the designated treatments, cells were washed with PBS. Then, 1 mL of serum-free DMEM and 1 mL of JC-1 staining solution were added to each well, ensuring thorough mixing. The plate was placed in a light-protected environment at 37 °C and incubated for 20 min. The supernatant was discarded, and the cells were washed twice with JC-1 staining buffer. Subsequently, 2 mL of serum-free DMEM was added. Changes in MMP were observed under a fluorescence microscope.

### 4.10. Enzyme-Linked Immunosorbent Assay (ELISA) for the Detection of HF Biomarkers and Associated Inflammatory Factors

After anesthetizing the rats, blood samples were collected from the abdominal aorta into blood collection tubes. Following natural coagulation at room temperature for 30 min, the tubes were centrifuged at 3000 rpm for 15 min to separate the serum. Then, the separated serum was subjected to the determination of NT-proBNP, TNF-α, and IL-1β levels in accordance with the instructions provided by the respective assay kits.

### 4.11. Histopathological Examination

After promptly extracting heart tissue upon blood collection, it was fixed with 4% paraformaldehyde, followed by paraffin embedding. The myocardial tissue was then sectioned into 5 μm slices. These sections underwent deparaffinization with xylene and ethanol, followed by staining with hematoxylin–eosin (HE) as well as Masson’s trichrome stain. Following the completion of staining, the sections were dehydrated with ethanol, made transparent with xylene, and mounted with neutral resin for microscopic examination. Images were captured and analyzed for histopathological evaluation.

### 4.12. Immunohistochemical Analysis

The cardiac tissues from rats were embedded in paraffin and sectioned, followed by deparaffinization in water. The sections were then placed in a retrieval box containing citrate antigen retrieval buffer (pH 6.0) and subjected to antigen retrieval in a microwave oven. After natural cooling, the slides were washed by shaking on a decolorizing shaker using PBS three times. A solution of 3% hydrogen peroxide was applied to the slides to block endogenous peroxidase activity. Following blocking, the slides were incubated overnight with the primary antibody, then washed and incubated for 50 min with HRP-conjugated secondary antibody. After thorough PBS washing, the slides were subjected to DAB staining, followed by counterstaining with hematoxylin to visualize the cell nuclei. Finally, the slides were dehydrated, mounted, and analyzed under a microscope.

### 4.13. Western Blot Analysis

The tissue samples were washed with pre-chilled PBS to remove blood contaminants, then cut into small pieces and placed into homogenization tubes. Two 4 mm homogenization beads and eight times the tissue volume of lysis buffer were added for homogenization. After homogenization, the samples were kept on ice for 30 min, with intermittent shaking every 5 min to ensure complete tissue lysis. Subsequently, the samples were centrifuged at 12,000 rpm for 10 min at 4 °C, and the protein supernatant was collected and its concentration determined using a BCA assay kit. The protein solution was mixed with sample buffer at a 4:1 ratio, subjected to denaturation in a boiling water bath, separated by 8%, 10%, and 12% SDS-PAGE gels, and electrophoresis was initiated once the bromophenol blue reached approximately 1 cm from the bottom of the gel. Electrophoresis was stopped, and the separated proteins were transferred onto a PVDF membrane. The membrane was placed in an incubation chamber containing TBST and incubated with 5% skim milk at room temperature for 30 min. Primary antibodies PGC1α (1:2000), PPARα (1:2000), and ACTIN (1:2000) were added and incubated overnight at 4 °C. After TBST washing, secondary antibodies (1:5000) were added and incubated for 30 min. The washed PVDF membrane was subjected to ECL chemiluminescence detection, and quantitative analysis and assessment were conducted using the AIWBwellTM 2.0 software.

### 4.14. Statistical Analysis

Statistical analysis was conducted using SPSS 25.0 software. Data from each group are presented as mean ± standard deviation. Comparisons between two groups were performed using the LSD-*t* test, while comparisons among multiple groups were analyzed using one-way analysis of variance (ANOVA). In cases of heterogeneity of variance, data transformation was applied. * *p* < 0.05 indicates a statistically significant difference, and ** *p* < 0.01 indicates an extremely significant difference.

## 5. Conclusions

In this study, our in vitro experiments demonstrated that GRb1@PLGA@NPs effectively reduce ROS generation, modulate oxidative stress-related indicators, and mitigate oxidative damage in H9c2 myocardial cells induced by hypoxia–reoxygenation. In vivo animal experiments further revealed that GRb1@PLGA@NPs improve pathological changes in HF rats, reducing the expression of NT-proBNP, TNF-α, and IL-1β in cardiac tissues. The cardioprotective and HF therapeutic mechanisms of GRb1@PLGA@NPs are likely associated with the modulation of the organismal energy metabolism through the ROS/PPARα/PGC1α pathway. To delve deeper into the effects of GRb1@PLGA@NPs on HF treatment, we will conduct subsequent experiments involving siRNA and the downstream transcription factors of the ROS/PPARα/PGC1α pathway for validation. Moreover, targeting strategies for GRb1@PLGA@NPs remain a focal point of our investigation.

## Data Availability

All data are contained within the article.
